# Cardiac Sparing with Personalized Treatment Planning for Early-stage Left Breast Cancer

**DOI:** 10.7759/cureus.7247

**Published:** 2020-03-12

**Authors:** Dominique Mathieu, Stéphane Bedwani, Julia Mascolo-Fortin, Nicolas Côté, Andrée-Anne Bernard, David Roberge, Michael Yassa, Houda Bahig, Toni Vu

**Affiliations:** 1 Radiation Oncology, University of Montréal Health Centre, Montréal, CAN; 2 Radiation Oncology, Maisonneuve-Rosemont Hospital, Montréal, CAN

**Keywords:** breast cancer, tangential intensity modulated radiotherapy, helical tomotherapy, deep-inspiration breath-hold, accelerated partial breast irradiation

## Abstract

Purpose

To compare cardiac doses of different whole-breast optimization schemes including free-breathing (FB) tangential radiotherapy (TRT), deep-inspiration breath-hold (DIBH) TRT, and FB helical tomotherapy (HT).

Methods

Early-stage left-sided breast cancer patients who underwent breast-conserving surgery followed by adjuvant radiotherapy were included in the study. Planning images included FB and DIBH CT scans acquired in the same supine treatment position with both arms abducted. A hypofractionated regimen of 42.5 Gy in 16 fractions was used. Clinical target volume delineation was aided through the use of a radio-opaque wire. A 7-mm margin was used in generating the planning target volumes. TRT plans were generated both in FB and DIBH. For the FB tomotherapy technique, a first plan (Tomo 1) was optimized limiting the maximum contralateral breast dose to 3.1 Gy. A second tomotherapy plan (Tomo 2) focused on the reduction of the mean heart dose without controlling the contralateral breast dose. All plans were optimized to obtain an equivalent planning target volume (PTV) coverage of ≥95% of the prescribed dose while minimizing the dose to organs at risk.

Results

Twenty-three patients treated between October 2012 and March 2016 were included in this retrospective study. Eleven patients (48%) had at least one major cardiovascular risk factors including one patient (4%) with a history of myocardial infarction. Six patients (26%) had been exposed to cardiotoxic chemotherapy agents. The average mean dose to the heart was 3.1 Gy with FB TRT, 1.1 with DIBH TRT, 2.4 Gy for Tomo 1, and 1.5 Gy for Tomo 2. The mean dose to the left anterior descending artery was 27.0 Gy, 8.0 Gy, 13.7 Gy and 6.6 Gy for FB TRT, DIBH TRT, Tomo 1 and Tomo 2 plans respectively.

Conclusion

Different cardiac-sparing optimization schemes are possible when treating left breast cancer. Although DIBH offers clear mean heart dose reductions, tomotherapy can be an interesting alternative treatment modality to spare the heart and coronary vessels, notably in patients who cannot comply with DIBH.

## Introduction

Adjuvant whole-breast radiotherapy following breast-conserving surgery significantly reduces locoregional recurrence and improves long-term survival [1]. However, breast irradiation carries an increased risk of major coronary events, which rises linearly with the mean dose to the heart [2]. It is therefore warranted to incorporate a concern for competitive risk of cardiovascular death into treatment decision making. Over the years, advances in photon-based radiotherapy such as personalized field-shaping using multileaf collimator (MLC), rotating gantry techniques, and flattening filter-free (FFF) beams have expanded the available techniques for personalized cardiac-sparing breast treatments. Additionally, improvement in image-guided radiation therapy (IGRT) and selection of patients amenable to more targeted radiotherapy have paved the way to much smaller irradiated volumes with less cardiac exposition.

Reduction of the irradiated cardiac volume can be accomplished through MLC in tangential radiotherapy (TRT), it often presents suboptimal heart doses in free-breathing (FB). Compared to FB TRT, deep-inspiration breath-hold (DIBH) TRT can be used to move the cardiac silhouette outside of the tangential fields by creating a separation between the heart and chest wall through lung inflation. Helical tomotherapy (HT) enables coverage of complex volumes with the excellent conformity of the dose distribution throughout a rotating gantry with treatment delivery from 360 degrees around the patient. Accelerated partial breast irradiation (APBI) is an approach that treats only the lumpectomy bed plus a 1-2 cm margin in patients aged 50 or older with early-stage invasive carcinoma as well as those with screen-detected carcinoma in situ.

In this study, we provide a comprehensive comparison of modern photon-based breast radiotherapy treatments with different cardiac-sparing approaches relying on delivery techniques, dosimetry optimization approaches, as well as a selection of low-recurrence risk patients amenable to reduced target volumes. The primary aim is to compare dosimetric variations to the heart and left anterior descending artery (LAD) for different whole-breast optimization schemes using FB TRT, DIBH TRT, and HT. Whole-breast results are compared to APBI plans for patients eligible for seroma-targeted irradiation.

## Materials and methods

Study population

Early-stage breast cancer patients who underwent breast-conserving surgery followed by adjuvant radiotherapy were included in this retrospective study. The study received institutional review board approval. Patients were selected according to the following criteria: (1) left-sided breast cancer; (2) pathologic confirmation of invasive carcinoma or carcinoma in situ; (3) partial mastectomy with or without axillary sampling; (4) scheduled for adjuvant tangential whole-breast radiation therapy; (5) nodal irradiation not indicated. The following cardiovascular risk factors were documented for each patient: past medical history of cardiac disease, hypertension, diabetes, hypercholesterolemia, obesity (BMI ≥30) and tobacco usage. Exposition to cardiotoxic systemic therapies was also reported. Patients eligible for APBI as per the consensus statement from the American Society for Radiation Oncology (ASTRO) were identified [3].

Radiation therapy planning

Planning images included a FB and a DIBH CT scan acquired without iodine injection in the same supine treatment position with both arms abducted above the head using 3-mm slices from mandible to diaphragm. DIBH acquisition was obtained with Abches (Apex Medical Inc. Tokyo, Japan) system, a breathing monitoring device with direct visual feedback that allows the patient to self-control the respiratory motion of the chest and abdomen [4]. Brief coaching was performed before DIBH CT acquisition to ensure that patients could maintain a reproducible breath-hold of approximately 20 seconds.

For the whole-breast plans, a radio-opaque wire was placed on the breast for clinical target volume (CTV) delineation and planning target volume (PTV) was obtained with a 7-mm expansion excluding 5 mm from the skin surface. All plans were optimized with a nominal energy of 6 MV to obtain an equivalent PTV coverage of ≥95% of the prescribed dose of 42.5 Gy in 16 fractions. The maximum tolerated point dose was 45.5 Gy. Constraints to organs at risk were as per the Radiation Oncology Therapy Group (RTOG) 1005 protocol [5]. For the FB and DIBH TRT plans, two conformal tangential fields and MLC were optimized field-in-field using inverse planning with a Varian 21EX linear accelerator (Varian Medical Systems, Palo Alto, CA) with a Millennium 80‐leaf MLC (Varian Medical Systems, Palo Alto, CA). For the tomotherapy technique, a first plan (Tomo 1) was optimized limiting the maximum contralateral breast dose to 3.1 Gy. A second tomotherapy plan (Tomo 2) focused on the reduction of the mean heart dose without controlling the contralateral breast dose to fully use the optimization and delivery capacity of the tomotherapy system.

For the APBI technique, plans were optimized using FB CT scans. Five non-coplanar intensity-modulated fields were used to obtain an equivalent PTV coverage of ≥95% of the prescribed dose of 30 Gy in 5 fractions. The CTV was defined as the surgical cavity plus 1 cm of surrounding breast tissue excluding the chest wall, pectoralis major, and skin. PTV was created through a 1-cm expansion of the CTV. Patients without a visible seroma on planning images were excluded from this analysis. Surgical clips delimiting the lumpectomy cavity were not used. Organs at risk doses were minimized as per the Once-a-Day Accelerated Partial Breast Irradiation (OPAR) protocol, with a focus on the reduction of the heart dose [6]. The analytical anisotropic algorithm on the Eclipse treatment planning system (Varian Medical Systems, Palo Alto, CA) was used for all dose calculations.

Structure delineation and dosimetric evaluation

The whole heart (WH) and LAD were delineated on DIBH and FB scans as per validated heart atlas [7]. To avoid interobserver variations, all structures were delineated by one observer only using a window level of 50 Houndsfield Units (HU) and a window with 500 HU. The WH was delineated from the inferior border of the left pulmonary artery to the apex and included all great vessels except the inferior vena cava. The LAD arteries were delineate using a 5-mm brush to account for motion uncertainties from the left side of the ascending aorta to the apex via the interventricular groove. Individual lungs and the contralateral breast were also contoured using RTOG atlas. Contours were performed by the same experienced radiation oncologist and validated by the physicist.

Comparisons of the following dosimetric values for all plans were performed: the mean heart dose, the volume (cc) of the heart receiving 20 Gy (V20) and 30 Gy (V30), the mean and maximal LAD doses, the mean left lung dose, the volume (%) of the left lung receiving 20 Gy (V20) and 5 Gy (V5), the right breast maximal dose, and the volume of the right breast receiving 3.1 Gy (V3.1). Dose results of all whole breast and APBI techniques were compared using equivalent dose in 2 Gray per fraction (EQD2) with the following formula: nd[(d+α/β)÷(2+α/β)], where n was the number of fractions, d was the dose per fraction (in Gy), and α/β was 3.

Statistics

A non-parametric Quade test was carried out to see if there were differences in cardiac dose based on four optimization schemes used for treatment planning. Considering four treatment types, the Quade test was expected to be more powerful than the Friedman's test [8]. The level of statistical significance was set to p-values below 0.05. A pairwise comparison using Quade post-hoc test was conducted after significant results. All statistical tests were performed in R (version 3.4.3, R Core Team, 2017) using the PMCMRplus package. A linear regression was conducted between the mean heart dose calculated from FB TRT plans and the other treatment plans to address cardiac dose sparing. Descriptive statistics were provided for both the patient cohort and the dosimetric results between whole and partial breast irradiation.

## Results

Twenty-three patients treated between October 2012 and March 2016 were included in this retrospective study. The median age was 54 years (range: 37-73 years). Eleven patients (48%) had at least one major cardiovascular risk factors including one patient (4%) with a history of myocardial infarction. Six patients (26%) had been exposed to cardiotoxic chemotherapy agents. Patient and treatment characteristics are summarized in Table 1.

**Table 1:** Patient and treatment characteristics (n = 23) IDC: invasive ductal carcinoma; DCIS: ductal carcinoma in situ; HR+: hormone receptor positive; HER2+: human epidermal growth factor receptor 2 positive

Patient characteristics	
Median age (range), years	54 (37-73)
Histology, n (%)	
IDC	15 (65)
DCIS	8 (35)
Tumour size (mm), n (%)	
≤10	12 (52)
10-20	9 (39)
20	2 (9)
Localization, n (%)	
Medial	7 (30)
Lateral	14 (61)
Other	2 (9)
Receptor status, n (%)	
HR+	19 (83)
HER2+	4 (17)
Triple-	2 (9)
Hormonotherapy exposure	18 (78)
Chemotherapy exposure, n (%)	
Anthracycline-based	3 (13)
Trastuzumab	3 (13)
Cardiac risk factors, n (%)	
Any risk factors	11 (48)
Ischemic heart disease	1 (4)
Hypertension	5 (22)
Diabetes	1 (4)
Hypercholesterolemia	3 (13)
Obesity	2 (8)
Tobacco usage	1 (4)

**Figure 1:**
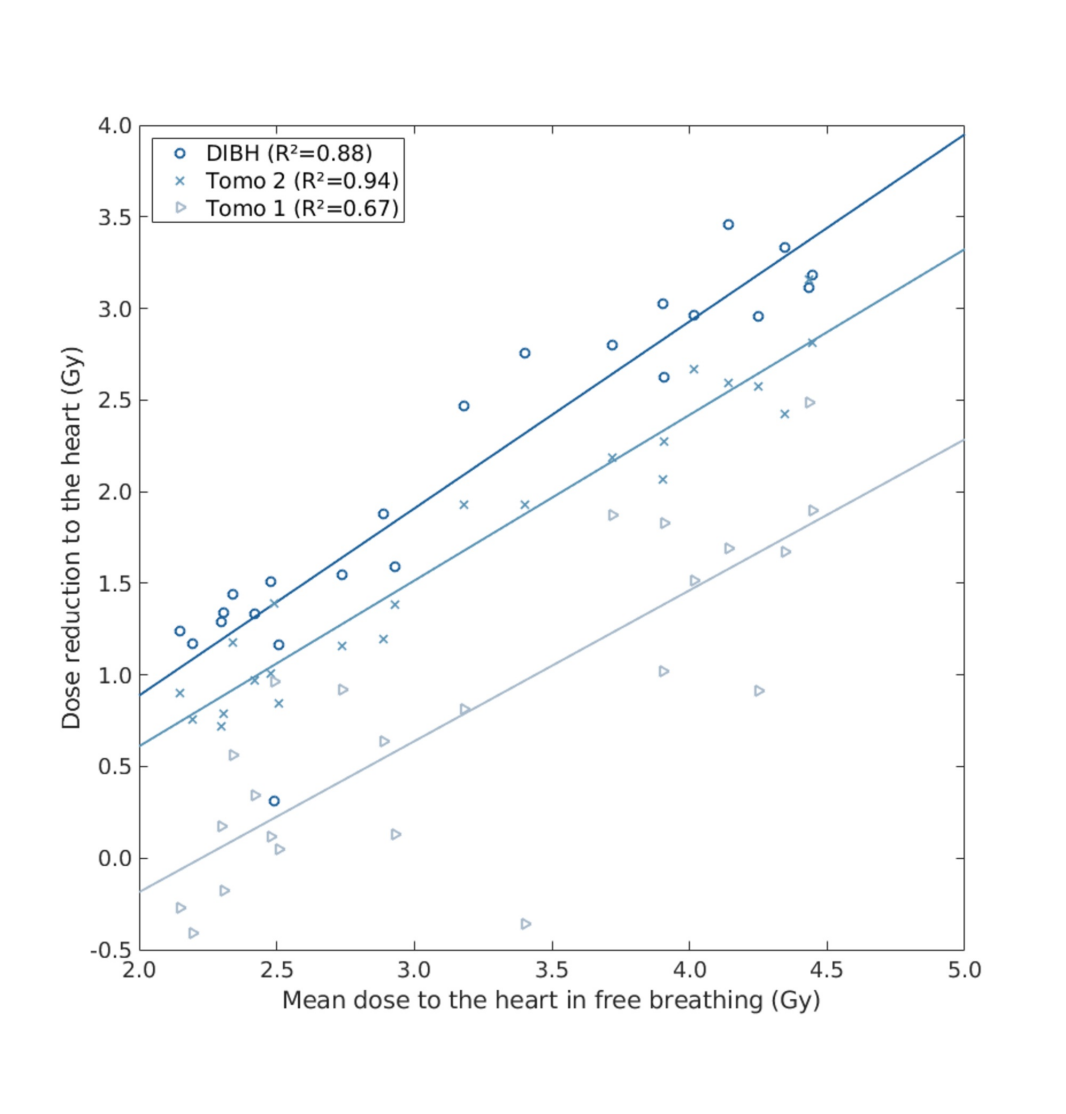
Mean heart dose reduction compared to free-breathing tangential radiotherapy plans DIBH: deep-inspiration breath-hold; Tomo: tomotherapy

For the whole-breast techniques, the average mean dose to the heart was 3.2 Gy with FB TRT, 1.1 Gy with DIBH TRT, 2.4 Gy for Tomo 1, and 1.5 Gy for Tomo 2. A graphical representation of the mean heart dose reduction compared to FB TRT is presented in Figure 1.

**Figure 2:**
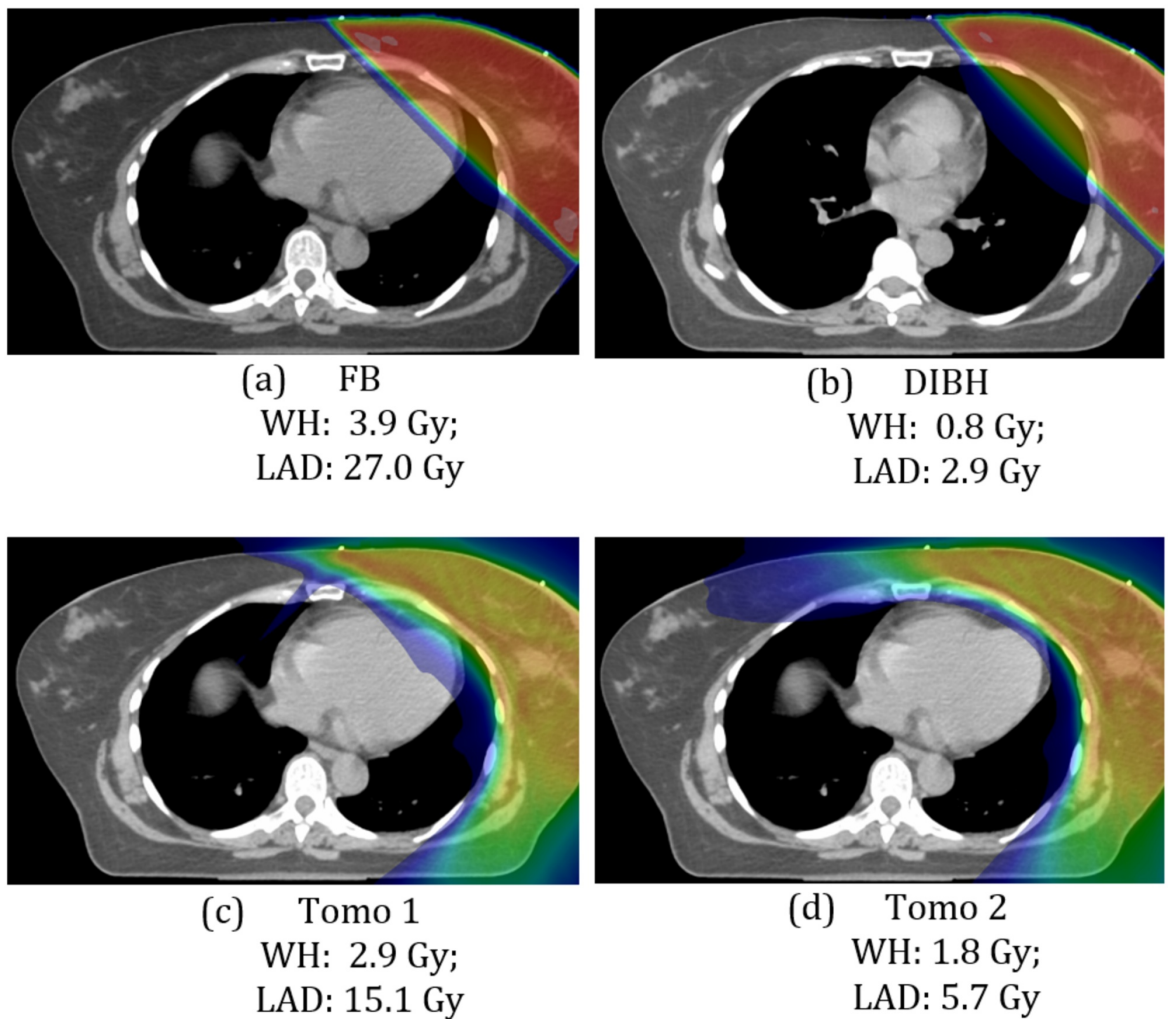
Typical color-wash dose distributions and mean cardiac doses of different whole breast treatment plans (a) free-breathing; (c) deep-inspiration breath-hold; (c) Tomo 1; (d) Tomo 2 FB: free-breathing; DIBH: deep-inspiration breath-hold; Tomo: tomotherapy; WH: whole heart; LAD: left anterior descending artery

The mean doses to LAD were 27.0 Gy, 8.0 Gy, 13.7 Gy, and 6.6 Gy for FB TRT, DIBH TRT, Tomo 1, and Tomo 2 plans respectively. Comparison of color-wash dose distributions and mean cardiac doses of all different whole-breast optimization techniques for a typical left breast case is presented in Figure 2.

Left lung mean dose and V20 remained constant for all plans. As expected, the average contralateral breast dose was significantly higher with the Tomo 2 plans at 17.6 Gy compared to less than 2 Gy with the other techniques. All dosimetric results are presented in Table 2.

**Table 2:** Dosimetric results for the whole-breast plans (n = 23; prescribed dose: 42.5 Gy/16 fx) SD: standard deviation; FB: free-breathing; DIBH: deep inspiration breath-hold; Tomo: tomotherapy; WH: whole heart; LAD: left anterior descending artery; V: percentage of volume receiving at least a given dose

	FB (mean ±SD)	DIBH (mean ±SD)	Tomo 1 (mean ±SD)	Tomo 2 (mean ±SD)
WH				
Max (Gy)	43.5 ±0.5	31.6 ±12.3	35.0 ±5.0	28.0 ±8.2
Mean (Gy)	3.2 ±0.8	1.1 ±0.3	2.4 ±0.5	1.5 ±0.2
V20 (%)	5 ±2	0.5 ±0.7	1 ±1	0.2 ±0.3
LAD				
Max (Gy)	42.8 ±1.0	22.8 ±14.2	28.7 ±5.8	20.1 ±7.6
Mean (Gy)	27.0 ±6.3	8.0 ±7.6	13.7 ±4.1	6.6 ±2.4
Left lung				
Mean (Gy)	6.6 ±2.3	6.2 ±1.8	5.9 ±1.1	4.3 ±0.8
V5 (%)	22 ±7	23 ±6	30 ±5	21.9 ±4.0
V20 (%)	13 ±6	12 ±4	8 ±4	5 ±2
Right breast				
Max (Gy)	1.3 ±0.2	1.9 ±0.8	3.4 ±0.6	17.6 ±4.6
V3.1 (%)	0.0 ±0.0	0.0 ±0.0	0.1 ±0.4	33 ±15

**Figure 3:**
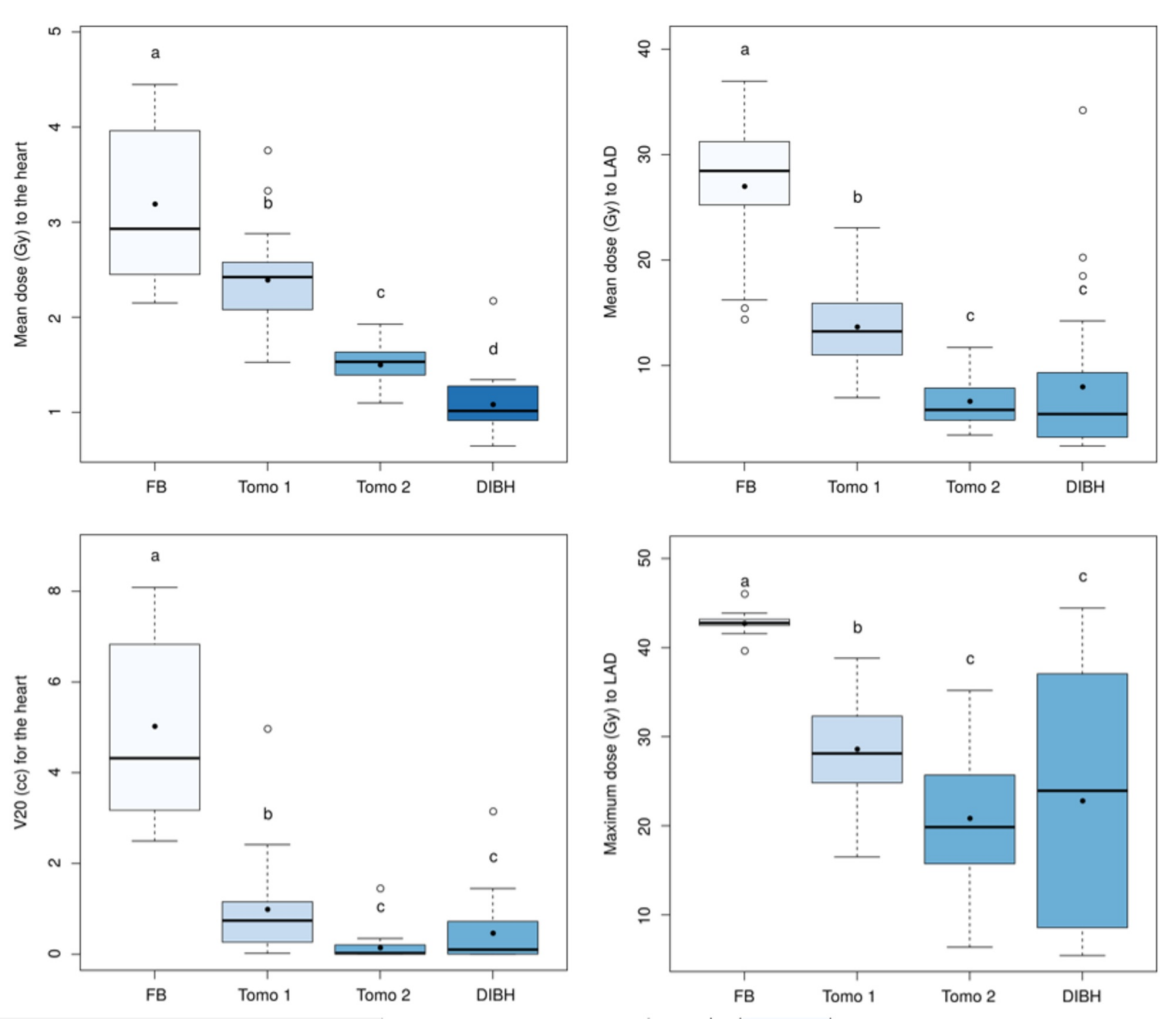
Box plot of the mean and maximum whole-breast plans cardiac doses The black dots and circles indicate respectively the mean and outlier values. The colors and corresponding group letters represent technic with statistically similar heart dose results FB: free-breathing; Tomo: tomotherapy; DIBH: deep-inspiration breath-hold; V20: percentage of volume receiving at least 20 Gy; LAD: left anterior descending artery

A Quade analysis of variance was applied for each of the following dosimetric parameters: V20 and mean dose to the heart; maximum and mean doses to LAD; and has indicated significant differences (p: <0.01 for all tests) depending on the treatment type (FB, Tomo 1 and 2, DIBH). The post-hoc analysis allowed us to rank each treatment according to cardiac dose sparing. Treatments were ranked as follows: FB <Tomo 1 <Tomo 2 <DIBH for the reduction of the heart mean dose and FB <Tomo 1 <Tomo 2, DIBH for the heart V20, as well as LAD mean and maximum doses. These statistical results are shown in Figure 3.

Eleven (48%) patients were eligible for seroma-targeted radiotherapy as per the updated ASTRO guidelines. Among them, nine patients had visible seroma on planning images and were subsequently included in the APBI plan analysis. Detailed results of the APBI plans are presented in Table 3.

**Table 3:** Dosimetric results for the accelerated partial breast irradiation plans (n = 9; prescribed dose: 30 Gy/5 fx) APBI: accelerated partial breast irradiation; SD: standard deviation; WH: whole heart; LAD: left anterior descending artery; V: percentage of volume receiving at least a given dose

APBI results	Mean ±SD
WH	
Max (Gy)	9.3 ±9.2
Mean (Gy)	0.5 ±0.3
V20 (%)	0.2 ±0.5
LAD	
Max (Gy)	7.2 ±9.6
Mean (Gy)	3.0 ±4.6
Left lung	
Mean (Gy)	1.4 ±0.6
V5 (%)	7 ±4
V20 (%)	0.8 ±1.1
Right breast	
Max (Gy)	0.3 ±0.3
V3.1 (%)	0.0 ±0.0

Mean heart and LAD doses were respectively 0.5 Gy and 3.0 Gy with APBI technique. As compared to FB TRT, mean heart dose was reduced by 1.8 Gy with APBI in equivalent dose in 2 Gy fractions (EQD2) as shown in Table 4.

**Table 4:** Equivalent dose in 2 Gy fractions results for whole-breast (42.5 Gy/16 fx) and accelerated partial breast irradiation plans (30 Gy/5 fx) SD: standard deviation; EQD2: equivalent dose in 2 Gy fractions; FB: free breathing; DIBH: deep inspiration breath-hold; Tomo: tomotherapy; APBI: accelerated partial breast irradiation; WH: whole heart; LAD: left anterior descending artery

EQD2 (Gy)	FB (mean ±SD)	DIBH (mean ±SD)	Tomo 1 (mean ±SD)	Tomo 2 (mean ±SD)	APBI (mean ±SD)
WH					
Max	49.7 ±1.4	33.3 ±15.4	36.6 ±7.4	27.5 ±10.9	12.0 ±18.3
Mean	2.1 ±0.6	0.7 ±0.2	1.5 ±0.3	0.9 ±0.1	0.3 ±0.2
LAD					
Max	48.6 ±1.8	22.6 ±17.0	27.9 ±7.7	18.7 ±8.7	9.7 ±18.0
Mean	25.8 ±7.7	6.3 ±7.7	10.8 ±4.0	4.6 ±1.9	2.9 ±5.7
Left lung					
Mean	4.6 ±1.7	4.2 ±1.3	4.0 ±0.9	2.9 ±0.6	1.0 ±0.5
Right breast					
Max	0.8 ±0.1	1.2 ±0.6	2.2 ±0.4	14.6 ±4.9	0.2 ±0.2

## Discussion

Breast radiation therapy carries a long-term risk of cardiac toxicity. The probability of major coronary events such as myocardial infarction, coronary revascularization, and death from ischemic heart disease increases linearly with the mean dose to the heart and LAD, with no minimum threshold for risk [2]. In conjunction with heart radiation dose, systemic agents, such as anthracycline-based chemotherapy, play a synergetic role in the development of cardiac dysfunction. The American Society of Clinical Oncology (ASCO) recommends usage of preventive strategies to minimize the risk of cardiotoxicity in specific breast cancer patients groups, including those exposed to (1) high-dose anthracycline (≥250 mg/m2 doxorubicin, ≥600 mg/m2 epirubicin), (2) high-dose (≥30 Gy) radiotherapy where the heart is in the treatment field, (3) lower-dose anthracycline in combination with lower-dose radiotherapy (<30 Gy) where the heart is in the treatment field, or (4) treatment with lower-dose anthracycline or trastuzumab alone and presence of pre-existing cardiovascular risk factors. In these high-risk patients, clinicians should use cardiac-sparing techniques with more conformal radiation fields as well as lower radiotherapy doses to reduce radiation-induced long-term cardiotoxicities [9]. In this study, we compared cardiac doses of different whole-breast optimization schemes including FB TRT, DIBH TR, tomotherapy, and seroma-targeted external beam photon with APBI.

Although reduction of the irradiated cardiac volume can be accomplished through personalized field-shaping using MLC, TRT often presents suboptimal heart doses in left-sided breast cancer patients. Compared to FB, DIBH TRT can be used to move the cardiac silhouette outside of the tangential fields by creating a separation between the heart and chest wall. In our study, we found a significant mean heart dose reduction using DIBH, with a mean heart dose of 1.1 Gy compared to 3.2 Gy with FB TRT. This 2 Gy reduction, which can be translated into a 14% relative risk reduction of a major coronary event as per Darby et al., is consistent with previous reports [10-11]. In a study including 30 patients with left-sided breast cancer treated with adjuvant breast radiotherapy of 50 Gy in 25 fractions, DIBH resulted in a significant reduction in mean heart and LAD doses of 3.0 Gy and 9.8 Gy respectively compared to FB treatments. Lee et al. also reported similar dose reduction results in 25 patients with left-sided breast cancer treated in DIBH using the Abches device, with a mean heart dose reduction of 2.0 Gy. Interestingly, this technique is both reproducible and can be implemented in a clinical setting at low cost, as reported by Conroy et al. [12], with its low-resource visually monitored DIBH technique that relies solely on skin markers. Although DIBH has proven to be an effective cardiac-sparing technique, this technique is not suitable for all patients as it requires a good pulmonary function to hold the breath for an approximate duration of 20 seconds, good collaboration and understanding of the directives, as well as anxiety management.

HT can be a cardiac-sparing alternative to DIBH. This technique enables coverage of complex volumes with the excellent conformity of the dose distribution. Compared to FB tangential results, HT resulted in a significant cardiac sparing with mean heart doses of 2.4 Gy with Tomo 1 and 1.5 Gy with Tomo 2 plans, results similar to those previously published [13-14]. In a study of 20 early-stage breast cancer patients that underwent breast-conserving surgery, FB tomotherapy resulted in a significant reduction of 0.6 Gy to mean heart dose as compared to FB TRT and could even be lowered by an additional 1.0 Gy when combining tomotherapy and DIBH, an approach that is indeed difficult to imagine in a clinical setting [14]. Compared to tangential technique, tomotherapy delivers a larger dose spread with increased low-dose areas, especially for organs not normally irradiated, such as the contralateral breast, and selection of optimization parameters and field blocking at specific angles help minimize the risk or late secondary malignancy. However, dosimetric optimization parameters can sometimes be individualized to patients, as concomitant cardiovascular risk factors may represent a greater competitive risk factor of death than secondary malignancy. Moreover, younger patients who had prophylactic bilateral mastectomy for breast cancer prevention could benefit from this optimization approach, as they may get a longer surviving time interval to develop such cardiac toxicity. In our study, a tomotherapy plan focusing on the reduction of the mean heart dose without controlling the contralateral breast dose resulted in a mean heart dose of 1.5 Gy, results clinically comparable to those obtained in DIBH.

ASTRO extended the early-stage breast cancer patients eligible to APBI in its updated 2017 clinical practice statement to include patients aged 50 or older with early-stage invasive carcinoma as well as those with screen-detected low-risk ductal carcinoma in situ. In our cohort, nine eligible (39%) patients had visible seroma on CT planning images and were planned with this technique. Doses to the heart were dramatically reduced, with averages for mean WH and LAD doses of 0.5 Gy and 3.0 Gy respectively, lower than any whole-breast photon-based optimization schemes. Compared to whole-breast radiotherapy, cardiac doses reduction with APBI could impact non-breast-cancer mortality, as postulated by the authors of the TARGIT-A trial [15]. In this randomized non-inferiority trial, 3,451 patients with early-stage invasive ductal carcinoma were randomized to APBI with intraoperative single-dose radiotherapy of 20 Gy to the surface of the tumor bed versus whole-breast RT (40-56 Gy with or without a boost of 10-16 Gy). At the 4-year follow-ups, there was no difference in breast cancer mortality between groups but there were significantly fewer non-breast-cancer deaths with APBI (1.4% vs 3.5% for whole-breast radiotherapy; p: 0·0086), attributable to fewer deaths from cardiovascular causes and other cancers. These results must be interpreted with caution as no formal heart dosimetric analysis was performed in this trial.

Interestingly, APBI dosimetric results are comparable to dose expected with whole-breast proton irradiation. In a cohort of 20 left breast cancer patients treated to 42.5 Gy in 16 fractions, whole-breast intensity-modulated proton therapy in FB resulted in a mean heart dose of 0.2 Gy and a maximum LAD dose of 4.5 Gy [16]. More and more proton therapy clinical trials are enrolling patients with breast cancer to assess whether a reduction in cardiac toxicity can be achieved with this technique [17]. Unfortunately, the control arms of many of these trials often rely on simple FB TRT and possibly overexpose patients to high cardiac doses that could be significantly reduced with more robust treatment techniques. We believe clinicians should systematically propose personalized photon-based treatment planning in all left breast cancer patients, as proposed by most recent guidelines.

Limitations of this study include its retrospective nature as well as its sample size. Concerning dosimetric analysis, we did not assess dose to specific cardiac substructures including the left and right cardiac main arteries as well as the left ventricle. We did not integrate the dosimetric effects of the boost dose to the lumpectomy cavity that can be delivered with both photon and electron and may contribute to cardiac toxicity. We could have performed a dosimetric comparison with novel radiation therapy techniques such as volumetric modulated arc therapy (VMAT). Finally, we did not measure the effect of cardiac or respiration motion in the dosimetric analysis in any of the plans. Dose to LAD has been shown to vary with cardiac motion [18].

## Conclusions

Different cardiac-sparing optimization schemes are possible when treating left breast cancer. While DIBH offers clear mean heart dose reductions, tomotherapy can be an interesting treatment modality to reduce major coronary vessels doses, especially when contralateral breast dose reduction can be omitted. When appropriate, APBI with targeted seroma irradiation should be considered as an efficient heart preserving strategy for photon radiotherapy.
